# Oral HPV Infection in Women with HPV-Positive Cervix Is Closely Related to Oral Sex

**DOI:** 10.3390/diagnostics13122096

**Published:** 2023-06-16

**Authors:** Maria Teresa Bruno, Sara Boemi, Giuseppe Caruso, Francesco Sgalambro, Salvatore Ferlito, Antonio Cavallaro, Maria Chiara Sudano, Marco Palumbo

**Affiliations:** 1 Department of General Surgery and Medical Surgery Specialties, Gynecological Clinic, University of Catania, Via S.Sofia 78, 95123 Catania, Italy; giu.caruso97@gmail.com (G.C.); mpalumbo@unict.it (M.P.); 2 Multidisciplinary Research Center in Papillomavirus Pathology, University of Catania, 95123 Catania, Italy; saraboemi@live.it (S.B.); f.sgalambro@policlinico.unict.it (F.S.); ferlito@unict.it (S.F.); chiarasudano@hotmail.it (M.C.S.); 3 Department of Medical, Surgical Sciences and Advanced Technologies “G.F. Ingrassia”, University of Catania, 95123 Catania, Italy

**Keywords:** oral HPV, OCSS, oral sex, 9-valent HPV vaccine

## Abstract

The oral transmission of HPV and, consequently, the risk of oral cancer has increased in the last years. Oral sex has often been implicated among the risk factors for oral HPV infections, however, there is still no consensus on these topics, nor on the relationship between genital and oral HPV infections. The present study aimed to evaluate the coexistence of papilloma virus, at the levels of the oral and genital mucosa, in women with a histologically confirmed HPV lesions (and a positive HPV test) at the genital level and a negative HPV control group. We also evaluated how some risk factors, such as smoking, the number of partners, age, and sexual habits can influence the possible presence of the virus itself in the oropharynx of the same women. In total, 117 unvaccinated women aged between 18 and 52 were enrolled. We found that the prevalence of oral HPV infection was high among the women with concomitant genital HPV infection (22%) compared to the HPV-negative women (0%), and the estimated odds ratio was 17.36 (95% CI: 1.02, 297.04). In none of the women with oral HPV did we find any relevant clinical lesions. The potential risk factors for HPV infections in the oropharynx and genitals were analyzed based on questionnaire responses. A multivariate analysis showed that genital HPV infections were significantly associated with a number of sexual partners > 10 (OR 138.60, 95% CI: 6.04–3181.30, *p* < 0.001), but the data also referred to having between 3–5 or 6–10 partners as being significant, as were a high level of education (OR 6.24, 95% CI: 1.67–4.23.26 *p* = 0.003), a frequency of sexual intercourse >10 (OR 91.67 95% CI: 3.20–2623.52, *p* = 0.004), oral sex (OR 6.16, 95% CI: 1.22–31.19, *p* = 0.014), and >20 cigarettes/day (OR 6.09 95% CI: 1.21–30.61, *p* = 0.014). Furthermore, being “separate” and having multiple sexually transmitted diseases were also significantly associated with genital HPV infection. In contrast, oral HPV infections were significantly associated with women aged 36 to 50 years (OR 27.38, 95% CI: 4.37–171.37; *p* = 0.000202) and oral sex (OR 95.5, 95% CI: 5.13–1782.75, *p* = 0.001126).Additionally, being separate, being cohabitant, lifetime sexual partners of >10, 3–5 lifetime sexual partners, <20 years of age, >10 sexual intercourse per month, occasional and regular anal sex, >20 cigarettes per day, a history of sexually transmitted disease (herpes and multiple), and having a history of genital warts were significant. Screening and early diagnosis are considered to be practically unfeasible for this category of cancer, given the lack of visible lesions; the 9-valent HPV vaccine remains the only means that could help to successfully counter the growing incidence of oral squamous cell carcinoma.

## 1. Introduction

Genital HPV infection is the most prevalent sexually transmitted infection in the world [1]. It is estimated that more than 75% of sexually active women contract the infection in their lifetime. In Europe and North America, its peak prevalence is recorded in young women under the age of 25, soon after the onset of sexual activity. In 80% of young women, within 18–24 months, there is a clearance of the virus; however, the persistence of high-risk genotypes identifies the women at risk of preneoplastic lesions that, if not diagnosed and treated in time, can progress to cervical carcinoma [2,3]. Papillomavirus also exerts its oncogenic capacity against other organs: the oropharynx, anus, vulva, and penis [4]. The cervix, palatine tonsil, and anal region are characterized by the presence of a squamocolumnar junction, the site of metaplasia, which creates a suitable habitat for the reproduction of papillomavirus; in fact, cervical cancer, anal cancer, and cancer of the oropharynx occur more frequently in the population at risk. In recent decades, cervical cancer has caused the death of many young women and only the activation of a nationwide screening system, to which the use of the HPV vaccine has been added, has lowered the mortality of cervical cancer by 70% (7.4 per 100,000) and the HPV morbidity in industrialized countries [5]. In developed countries, oral squamous cell carcinoma (OSCC) is now the most frequent HPV-related cancer, having surpassed cervical cancer [6,7,8] and, by 2030, most squamous cell carcinomas will be HPV-related in comparison to the non-HPV-related form [9]. Oral HPV infection overtook smoking and alcohol as the main risk factor for oropharyngeal cancers [10,11]. The latter are more frequent in males [9], but lately, the female population has also seemed to show an increase in its incidence. 

The papillomavirus also seems capable of infecting and replicating in the cells of the syncytiotrophoblast, causing adverse events in pregnancy (abortion, gestosis, and preterm delivery) [12,13].

There are differences in the natural history of HPV based on gender and the anatomical site of infection. More HPV-related cancers are diagnosed worldwide in women than men, and HPV transmission appears to be higher in women than men [14]; moreover, the natural history of HPV infection in the cervix is well known, while that in the oropharynx is not entirely clear. The prevalence of HPV in the oropharynx is unknown, but published studies have found wide-ranging prevalence estimates from 2.6% to 50% [15,16,17], also producing conflicting results regarding the rate of women presenting the same type of HPV at two mucosal sites, as detected in 0–60% of cases. The route of transmission is unclear and the time until the elimination of HPV from the oropharynx is unknown, as the rate of viral clearance appears to be highly variable between studies, with a median time until elimination ranging from 6 to 18 months [18,19]. A question arises as to whether oral infection can develop from genital HPV infection, through oral and genital contact or by self-inoculation, or whether it should be considered as an independent event [4,8]. Relevant to this problem is the frequency of oral HPV infections in women with a cervical HPV infection. Oral sex has often been implicated among the risk factors for oral HPV infections [20], however, there is still no consensus on these topics, nor on the relationship between genital and oral HPV infections.

The main objective of our study was to evaluate the coexistence of papilloma virus at the levels of the oral and genital mucous in women with an HPV lesion at the genital level (positive HPV test) and a negative HPV control group. We also evaluated how some risk factors, such as smoking, number of partners, age, and sexual habits can influence the possible presence of the virus itself in the oropharynx of the same subjects.

## 2. Materials and Methods

The study sample consisted of 347 women who came to our HPV center for a suspected HPV infection in the period between March 2021 and October 2022. Ours is a multidisciplinary HPV Unit providing gynecological, proctological, and otolaryngologic screening for women or couples. In each clinic, swabs were used to search for the virus, in addition to diagnostic tests, such as colposcopy, peniscopy, an exploration of the oropharynx, laryngoscopy, and anoscopy.

The women underwent an inspection of their oropharynx, performed by an ENT specialist looking for relevant clinical lesions of the oropharynx. A cytobrush was then used to sample the oropharynx (soft palate, base of the tongue, and tonsils). The cytobrush was swirled vigorously in a PreservCyt vial. Immediately afterwards, the gynecologist took a sample of cells from the portio and cervical canal using an Ayre’s spatula and cytobrush, and the sample was transported into a thin prep container for papillomavirus detection and genotyping. In addition, after receiving their informed consent in writing, we gave each woman a detailed anonymous questionnaire (Supplementary File) in order to gather information on their age, smoking, and sexual habits. All these data are stored in the database of the multidisciplinary HPV center. Of the 347 women considered, 55 were excluded because they did not give their consent, 37 because the result of the HPV test was ambiguous, and 25 because they did not undergo the examination by the ENT specialist. Of the 130 remaining women, only 117 submitted the questionnaire; thus, the study population was 117 women, 87 that were genital HPV positive (study group) and 30 women that were genital HPV negative (control group). 

Concomitant infections were defined as any HPV infection that occurred simultaneously in both the oral cavity and cervix.

Concordant infections were identified in women who had at least one or more identical HPV types in the oropharynx and cervix, either at the same time or at different times.

The term oropharynx refers to the anatomical region comprising the soft palate and uvula, the tonsils, the posterior pharyngeal wall, and the base of the tongue. This region is distinct from the oral cavity, which includes the lips, the floor of the mouth, the buccal mucosa, the gum, the hard palate, and the mobile part of the tongue. Oral cancer refers to the combination of oropharyngeal and oral cancers.

### 2.1. DNA Extraction

An automated DNA extraction was carried out using the NucliSENS EASYMAG system (bioMérieux SA, Marcy l’Etoile, Lione, France), following the manufacturer’s HPV 1.1 protocol.

The DNA was extracted from the cell suspensions as described above [21].

### 2.2. HPV Genotyping

The kit used for the genotyping was Ampliquality HPV-TYPE EXPRESS (AB ANALITICA srl, Padova, Italy), which is able to determine the presence of 40 types of Papillomavirus, in particular: 16, 18, 31, 33, 35, 39, 45, 51, 52, 56, 58, 59, 68, 73, 82, 26, 53, 66, 67, 6, 11, 40, 42, 43, 61, 69, 70, 44, 54, 55, 62, 64, 71, 72, 81, 83, 84, 87, 89, and 90. 

The kit is based on a rapid system for the identification of human Papillomavirus using a single-step PCR: in the preparation of the PCR session, in addition to the samples, a positive amplification and typing control (HPV 61 DNA), as well as a negative control (sterile distilled water), were also used; in addition, the kit can evaluate the suitability of the DNA extracted via the amplification of the TST gene (thiosulfate sulfurtransferase rhodanese). For details of the technique, see [22].

### 2.3. Statistical Analysis

The statistical analyses were performed with SPSS version 22 for Windows. The Cohen kappa value was calculated to verify the concordance of HPV infections between the paired samples of the same patients. We evaluated the association between oral and genital HPV infections with a binary logistic regression analysis. To investigate the associations between the potential risk factors and HPV infections, we performed a multivariate analysis with a gradual binary logistic regression model. In cases where a frequency of 0 appeared, a correction was applied by adding 0.5. *p* values of <0.05 were considered to be statistically significant.

## 3. Results

The study group, consisting of women with an HPV-positive cervix, had a positive oral mucosal HPV of 21.8% (19/87). In none of the oral samples taken from the subjects of the control group was the presence of HPV DNA detected in the oropharynx. For oral HPV infection, the estimated odds ratio was 17.36 (95% CI: 1.02–297.04) (Table 1).

In particular, 21.8% of women had concomitant HPV infections, while concordant infections were found in 31.6% of women (6/19) (HPV 16, HPV 16, HPV 31, HPV51, HPV 6, and HPV 58). The concordance between genotypes was poor (kappa = 0.125).

Among the samples of the 87 women with HPV infections, we detected 121 HPVs (102 in genital samples and 19 in oral samples), 9 of which were high-risk HPVs and 14 were low-risk HPVs; the most common high-risk genotype was HPV16 (27.5%, 17), followed by HPV31 (12.6%, 7), and genotypes 33, 51, 52, and 58 with 5 cases each, followed by 45 and 68 with 1 case each.

Of the 19 women with oral HPV infections, 13 (68.4%) were infected with high-risk HPV and 6 (31.6%) with low-risk HPV. HPV16 was more common in the oral cavity (42.1%, 8/19).

In the women with cervical HPV lesions, genital warts accounted for 36.7% of cases (32 cases), low-grade squamous intraepithelial lesions for (LSIL) 33% (29 cases), and high-grade squamous intraepithelial lesions (HSIL) for 30% (26 cases). In the oral examinations, no relevant clinical lesions were found (subclinical infection).

The potential risk factors for HPV infections in the oropharynx and genitals were analyzed based on the questionnaire responses. A multivariate analysis showed that genital HPV infections were significantly associated with a number of sexual partners > 10 (OR 138.60, 95% CI:, 6.04–3181.30, *p* < 0.001), but the data also referred to having between 3–5 (OR 8.51, 95% CI: 1.93–37.45, *p* = 0.002) or 6–10 partners (OR 85.40, 95% CI: 10.02–727.54, *p* = 0.002 2.36216 × 10^−5^) as being significant, as were a high-grade education (OR 6.24, 95% CI: 1.67–4.23.26 *p =* 0.003) and a frequency of sexual intercourse > 10 (OR 91.67 95% CI: 3.20–2623.52, *p* = 0.004). In addition, a frequency of 5–10 (OR 15.40, 95% CI: 2.29–103.78, *p* = 0.002) sexual intercourse, oral sex (OR 6.16 CI 95%: 1.22–31.19, *p* = 0.014), and >20 cigarettes/day (OR 6.09 CI 95%: 1.21–30.61, *p* = 0.014) were significant. Furthermore, being “separate” (OR 51.72, 95% CI: 3.02–885.60, *p* = 0.003) and having multiple sexually transmitted diseases were also significantly associated with genital HPV infection. (Table S1) In contrast, oral HPV infections were significantly associated with advanced age (OR 27.38, 95% CI: 4.37–171.37; *p* = 0.000202) and oral sex (OR 95.5, 95% CI: 5.13–1782.75, *p* = 0.001126), but also with being separate, being cohabitant, lifetime sexual partners of >10 (OR 26.07, 95% CI: 1.30–521.94; *p* = 0.016483), 3–5 lifetime sexual partners, being <20 years of age (OR 5.13, 95% CI: 1.67–15.71; *p* = 0.002108), >10 sexual intercourse per month (OR 77.00, 95% CI: 2.67–2223.04; *p* = 0.005675), occasional anal sex (OR 18.29, 95% CI: 4.55–73.49; *p* = 2.11 × 10^−5^), regular anal sex (OR 28.44, 95% CI: 4.29–188, 75; *p* = 0.000263), >20 cigarettes per day (OR 7.75, 95% CI: 1.54–39.12; *p* = 0.006586), a history of sexually transmitted disease (herpes and multiple), and having a history of genital warts (OR 15.30, 95% CI: 3.34–70.12; *p* = 0.000222), (Table 2).

## 4. Discussion

The oral–genital transmission of HPV has the potential to affect the intimate life of women and their partners and has not often been explored in the literature. In our survey of 347 women, the collection of data, even if anonymously, was difficult and only 117 women (34%) answered the study questionnaire completely. The remainder either refused to participate or did not answer all the questions, invalidating their responses.

The oral transmission of HPV and, consequently, the risk of oral cancer is increased in women with cervical cancer and their spouses [23,24]; this finding suggests cross-transmission between the mouth and the genitals. In this study, one of the objectives was to compare the prevalence and concordance of HPV infections between oropharynx and genital sites in Italian women. We found that the prevalence of oral HPV infections was high among women with concomitant genital HPV infections (22%) compared to HPV-negative women (0%), and the estimated odds ratio was 17.36 (95% CI: 1.02–297.04). Thus, the number of women presenting with oral HPV also being positive for genital HPV is 17 times the number of women positive for oral HPV when the patient is negative for genital HPV. This is significant.

In the literature, the overall prevalence of oral HPV infections is varied, with values between 0.2% and 20.7% [25,26], and the rates of concomitant infections with oral and cervical HPV are less than 10%. Only a few studies [27,28] have shown concordant oral and cervical HPV infection rates greater than 65%. The variability in these data is due to the use of different inclusion criteria, clinical contexts, sampling methods, and detection tests. HPV16 was the most common high-risk genotype at both sites. In addition, we observed a low concordance between the HPV genotypes at the two anatomical sites in our cohort (kappa = 0.125).

In none of the women with oral HPV did we find any relevant clinical lesions. Therefore, an oral examination alone cannot exclude the possibility of an oral HPV infection, which is primarily subclinical.

The HPV-negative women in our study did not experience oral infection. Recent studies on healthy adults have found an oral prevalence of high-risk HPV strains of 2.5 to 5% [29,30].

The acquisition of HPV at any anatomical site can occur through an infected partner, however, it is difficult to distinguish which sexual behaviors are responsible for the transmission of HPV from the genital tract to the mouth.

In our study, the multivariate analysis showed that oral infection in women with HPV-positive genitals was significantly related to age and sexual habits, especially oral sex. If we look at the age of the women in the sample, we observe a statistically significant odds ratio of 27.38 (95% CI: 4.37–17.37) for women aged 36 to 50 years compared to patients aged less than 25 years. For the other classes, no statistically significant values were obtained.

With regard to sexual habits, the determinants most related to the detection of oral HPV are a history of STD and genital warts, the frequency of sexual intercourse, and the habit of practicing oral and anal sex. Specifically, the probability of women who had a history of sexually transmitted diseases to present an HPV infection at the oral level was 9.47% higher than that of the women who had never had a sexually transmitted disease (95% CI: 3.05–29.34). If genital herpes is considered, the odds ratio doubles from 9.47 to 19.88 (95% CI: 3.10, 127.60). 

Even with respect to the frequency of sexual intercourse, OR values greater than 1 that were statistically significant were obtained. The odds of being positive for oral HPV increased by 77% if the women had sexual intercourse more than 10 times per month (95% CI: 2.67, 2223.04), compared to those who had 0 or 1.

A very significant result was found with regard to the habit of anal sex: in women who practiced it “occasionally”, there was an increase of 18.29% in the odds of being positive for oral HPV, compared to those who did not practice it (95% CI: 4.55–73.49; *p*-value: 2.11 × 10^−5^). The odds ratio went up to 28.44 for those who practiced it regularly. Another risk factor detected in our sample, as in the case of genital HPV, seemed to be practicing oral sex regularly, compared to not practicing it at all, with an odds ratio of 95.59 (95% CI: 5.13–1782.75).

There was a strong association between oral sex and oral HPV. Hemminki K et al. suggested a major responsibility of oral sex as a cause of oral HPV, given that husbands of women with cervical cancer have a double risk of tonsillar cancer. Female genitalia have a higher transmission rate than male genitalia due to the keratinized penile epithelium, which is more resistant to HPV infection [14,31]. Even the amount of biological fluid that reaches the oropharynx can be contagious. Finally, it appears that women develop a stronger systemic immune response than men after a genital HPV infection. This would more efficiently protect women than men in the case of subsequent HPV exposure [32].

A multicenter study revealed a higher HPV detection rate in oral cancer samples among subjects who had oral sex habits and/or were sexually promiscuous [21]. Other ways of oral HPV contamination can be a self-injection and infection of the partner by transmission between the genitals, anal canal, hands, and oropharynx.

Our study highlighted that women with a previous histopathological diagnosis of cervical HPV were at a high risk of contracting subclinical oral HPV, as indicated by the presence of the virus in the oropharynx of 22% of patients, which was closely related to oral sex.

Screening and early diagnoses (absence of clinically visible lesion) are considered to be practically unfeasible for this cancer category, so primary prevention remains the only way to counteract the growing incidence of OSCCs. The use of condoms is widely accepted by both partners in vaginal intercourse, but their use appears to be very limited in oral–genital intercourse. It seems that the male population is not yet aware that oropharyngeal HPV infection is more dangerous than genital infection. 

Prophylactic HPV vaccines are effective in preventing HPV infection and HPV-associated cancers, including HPV-related OSCCs. The available epidemiological data report on the efficacy of the HPV vaccine in the prevention of oral infections [33,34,35,36].

HPV vaccination is currently the most promising prevention option for stemming the growing wave of HPV-positive oropharyngeal cancers in men. The lack of oral cancer precursor lesions to be used as an efficacy endpoint for OSCCs has delayed the approval of the HPV vaccine in males for oral cancer prevention; only since 2020 have the FDA in the United States and the EMA in Europe opened up to the use of the 9-valent vaccine also for males, with the specific indication of preventing oral cancer with the use of persistent viral infection as an endpoint for the risk of OCSSs [37,38]. More time is needed to investigate the benefit of HPV vaccines for OCSSs, given the long interval between HPV infection and the development of oropharyngeal cancers.

A limitation of this study could be its small sample size and the HPV sampling method. In our study, cytobrush test kits were used to collect superficial and deep samples of squamous cells from the oropharynx of the patients. Furthermore, in the literature, there is no agreement on sampling techniques and the best sampling method has yet to be identified. Oral HPV testing is not standardized and there are also no validated tests for assessing oral HPV in a clinical setting [31]. Another limitation of the study is the absence of reliable data on the clearance of the virus from the epithelium of the oropharynx. In fact, it is not possible to establish whether the negativity for HPV in the oropharynx should be attributed to the real absence of infection or to epithelial self-clearance that was able to eliminate the virus. Another bias is represented by the difficulty of the data collection, which, even if anonymously, was difficult, with only 34% of the women interviewed giving complete answers to the questionnaire and the rest refusing to or not answering completely. The use of questionnaires is subjective to the “recall bias” of the participants, which is caused by differences in the accuracy or completeness of the recollections retrieved by the study participants. Researchers should be aware of this. Condom use during oral sex and potential self-injection were also not examined, and the failure to analyze these data may create potential bias.

## 5. Conclusions

It is now commonly accepted that a subclinical oral HPV infection that persists for 10–30 years is an obligate precursor to most OSCCs [39,40,41]. Our results, in agreement with the studies cited, highlight the need for oral screening tests in women with high-risk cervical HPV lesions. However, our experience has shown that, even when persistent infection with an oncogenic type of HPV is demonstrated in the oropharynx, a clinically visible lesion is unlikely to be found as in the cervix [42,43,44]. Therefore, the conditions for effective and cost-effective oral HPV screening capable of identifying a preneoplastic lesions in the general population are not met. We believe it is necessary to recommend preventive measures, suggest changes in sexual behavior, and inform partners of the risk of oral cancer.

Considering that the adoption of barriers in orogenital intercourse is much more limited than vaginal intercourse, today, the 9-valent vaccine remains the only means to successfully counteracting the increasing incidence of OSCCs.

## Figures and Tables

**Table 1:** Estimated odds ratio of study group and control group for oral HPV.

	N°	Oral HPV+	Oral HPV−	OR	95% CI	*p*-Value
genital HPV−	30	0.5	30.5	1.00		
genital HPV+	87	19.5	68.5	17.36	1.02–297,04	0.024397

**Table 2:** Odds ratio related to risk factors for oral HPV infection.

Characteristics	N°	Oral HPV+	Oral HPV−	OR	95% CI		*p*-Value
Age (*n* = 117)							
18–25	38	1.5	37.5	1.00			
26–35	49	7.5	42.5	4.41	0.72	26.88	0.053728
36–50	21	11.5	10.5	**27.38**	**4.37**	**171.37**	**0.000202**
50+	9	0.5	9.5	1.32	0.05	34.93	0.434846
Marital Status (*n* = 117)							
Married	55	2.5	52.5	1.00			
Separate	38	12.5	25.5	**10.29**	**2.44**	**43.36**	**0.000742**
Cohabitant	22	5.5	16.5	**7.00**	**1.42**	**34.47**	**0.008365**
Divorced	2	0.5	1.5	7.00	0.22	218.96	0.133983
Education (*n* = 117)							
Middle school	18	2.5	16.5	1.00			
High school	49	8.5	41.5	1.35	0.30	6.19	0.34886
Graduate	44	9.5	35.5	1.77	0.39	8.00	0.230248
Academic Title	6	0.5	6.5	0.51	0.02	12.08	0.337521
Employed (*n* = 117)							
Engaged	38	5	33	1.00			
Student	35	8	27	1.96	0.57	6.67	0.142145
Self-employed	18	2	16	0.83	0.14	4.72	0.414473
Unemployed	26	4	22	1.20	0.29	4.97	0.400735
Age at first intercourse (*n* = 117)							
14–16 years old	43	4	39	1.00			
17–19 years old	59	8	51	1.53	0.43	5.45	0.2561
>20 years	15	3	12	2.44	0.48	12.45	0.142126
Number of lifetime sexual partners (*n* = 117)							
0–2	12	0.5	11.5	1.00			
3–5	58	4.5	53.5	1.93	0.10	38.49	0.332687
6–10	31	7.5	23.5	7.34	0.38	140.02	0.09256
>10	16	8.5	7.5	**26.07**	**1.30**	**521.94**	**0.016483**
Number of lifetime sexual partners <20 years of age (*n* = 117)							
0–2	84	9	75	1.00			
3–5	21	8	13	**5.13**	**1.67**	**15.71**	**0.002108**
6–10	12	2	10	1.67	0.31	8.84	0.274198
>10	0	0.5	0.5	8.33	0.16	446.20	0.148239
Frequency of sexual intercourse for month (*n* = 115)							
0–1	6	0.5	5.5	1.00			
2–4	33	3.5	29.5	1.31	0.06	28.97	0.433152
5–10	64	6.5	57.5	1.24	0.06	25.14	0.443517
>10	12	10.5	1.5	**77.00**	**2.67**	**2223.04**	**0.005675**
Oral sex (*n* = 109)							
Never	32	0.5	32.5	1.00			
Occasionally	57	7.5	50.5	9.65	0.53	174.83	0.062484
Regularly	20	12.5	8.5	**95.59**	**5.13**	**1782.75**	**0.001126**
Anal sex (*n* = 100)							
Never	67	3	64	1.00			
Occasionally	26	12	14	**18.29**	**4.55**	**73.49**	**2.11 × 10^−5^**
Regularly	7	4	3	**28.44**	**4.29**	**188.75**	**0.000263**
Smoke (*n* = 117)							
Never	33	2	31	1.00			
1–10 cigarettes a day	25	6	19	4.89	0.89	26.77	0.033479
11–20 cigarettes a day	29	1	28	0.55	0.05	6.44	0.318369
>20 cigarettes/day	30	10	20	**7.75**	**1.54**	**39.12**	**0.006586**
History of sexually transmitted diseases (STDs) (*n* = 111)							
No	76	5	71	1.00			
Yes	35	14	21	**9.47**	**3.05**	**29.34**	**4.92 × 10^−5^**
Chlamydia trachomatis	2	0.5	1.5	4.73	0.17	131.80	0.179845
Genital herpes	6	3.5	2.5	**19.88**	**3.10**	**127.60**	**0.000811**
Multiple sexually transmitted diseases	27	16.5	10.5	**22.31**	**6.77**	**73.50**	**1.65 × 10^−7^**
History of genital warts (*n* = 117)							
No	65	2	63	1.00			
Yes	52	17	35	**15.30**	**3.34**	**70.12**	**0.000222**

Statistically significant odds ratios are shown in bold.

## Data Availability

Data will be made available on request.

## Supplementary Materials

The following supporting information can be downloaded at: https://www.mdpi.com/article/10.3390/diagnostics13122096/s1, Table S1. Odds Ratio (OR) related to risk factors for genital HPV infection. Supplementary File: Anonymous questionnaire.
