# Supplementary material for: Oral HPV Infection in Women with HPV-Positive Cervix Is Closely Related to Oral Sex
Source: Diagnostics (Basel). 2023 Jun 16;13(12):2096. doi: 10.3390/diagnostics13122096 (PMC10297673; doi:10.3390/diagnostics13122096)
Supplement: Supplementary file 1 [file diagnostics-13-02096-s001.zip › Supplementary File Anonymous questionnaire.pdf]

Questionnaire

Answer the following statements by selecting a single answer, ticking the corresponding box with a cross

1. **Age**

- o 18-25
- o 26-35
- o 36-50
- o 50+

2. **Marital Status**

- o Married
- o Separate
- o Cohabitant
- o Divorced

3. **Education**

- o Middle school
- o High school
- o Graduate
- o Accademic Title

4. **Employmed**

- o Employee
- o Student
- o Self-employed
- o Unemployed

5. **Age at first intercourse**

- o 14-16 years old
- o 17-19 years old
- o >20 years

6. **Number of lifetime sexual partners**

- o 0–2
- o 3–5
- o 6–10
- o >10

7. Number of lifetime sexual partners <20 years of age

- 0–2
- 3–5
- 6–10
- >10

8. Frequency of sexual intercourse for month

- 0–1
- 2–4
- 5–10
- >10

9. Oral sex

- Never
- Occasionally
- Regularly

10. Anal sex

- Never
- Occasionally
- Regularly

11. Smoke

- Never
- 1-10 cigarettes a day
- 11-20 cigarettes a day
- >20 cigarettes /day

12. History of sexually transmitted diseases (STD)

- no
- yes
- Clamidia trachomatis
- herpes genitale
- Multiple sexually transmitted diseases

13. History of genital warts

14. no
15. yes
