# Supplementary material for: Oral HPV Infection in Women with HPV-Positive Cervix Is Closely Related to Oral Sex
Source: Diagnostics (Basel). 2023 Jun 16;13(12):2096. doi: 10.3390/diagnostics13122096 (PMC10297673; doi:10.3390/diagnostics13122096)
Supplement: Supplementary file 1 [file diagnostics-13-02096-s001.zip › diagnostics-2424123-Table S1.pdf]

Table S1: Odds Ratio (OR) related to risk factors for genital HPV infection.

Characteristics	N°	%	HPV+	OR	95% CI		p-value
**Age (n=117)**							
**18-25**	38	32,48%	30	1,00			
**26-35**	49	41,88%	37	0,82	0,30	2,27	0,353
36-50	21	17,95%	16	0,85	0,24	3,04	0,403
50+	9	7,69%	4	0,21	0,05	0,98	0,024
**Marital Status(n = 117)**							
**Married**	55	47,01%	33,5	1,00			
**Separate**	38	32,48%	38,5	**51,72**	**3,02**	**885,60**	**0,003**
**Cohabitant**	22	18,80%	14,5	1,15	0,42	3,12	0,395
**Divorced**	2	1,71%	2,5	3,36	0,15	73,29	0,221
**Education (n = 117)**							
**Middle school**	18	15,38%	10	1,00			
**High school**	49	41,88%	36	2,22	0,72	6,83	0,083
**Graduate**	44	37,61%	39	**6,24**	**1,67**	**23,26**	**0,003**
**Accademic Title**	6	5,13%	2	0,40	0,06	2,77	0,177
**Employmed status (n = 117)**							
**Engaged**	38	32,48%	28	1,00			
**Schoolgirl**	35	29,91%	33	**5,89**	**1,19**	**29,17**	**0,015**
**Freelancer**	18	15,38%	5	**0,14**	**0,04**	**0,48**	**0,001**
**Unemployed**	26	22,22%	21	1,50	0,45	5,05	0,256
**Age at first intercourse (n = 117)**							
**14-16 years old**	43	36,75%	31	1,00			
**17-19 years old**	59	50,43%	51	2,47	0,91	6,71	0,038
**>20 years**	15	12,82%	5	**0,19**	**0,05**	**0,68**	**0,005**
**Number of lifetime sexual partners (n = 117)**							
**0–2**	12	10,26%	2,5	1,00			
**3–5**	58	49,57%	39,5	**8,51**	**1,93**	**37,45**	**0,002**
6–10	31	26,50%	30,5	**85,40**	**10,02**	**727,54**	**2,36216E-05**
>10	16	13,68%	16,5	**138,60**	**6,04**	**3181,30**	**0,001**
**Number of lifetime sexual partners <20 years of age (n = 117)**							
**0–2**	84	71,79%	66	1,00			
**3–5**	21	17,95%	13	0,44	0,16	1,23	0,060
6–10	12	10,26%	8	0,55	0,15	2,02	0,182
>10	0	0,00%	0,5	0,27	0,01	14,23	0,260
**Frequency of sexual intercourse for month (n = 115)**							
**0–1**	6	5,22%	1,5	1,00			
**2–4**	33	28,70%	20,5	5,57	0,81	38,43	0,041

5–10	64	55,65%	52,5	**15,40**	**2,29**	**103,78**	**0,002**
>10	12	10,43%	12,5	**91,67**	**3,20**	**2623,52**	**0,004**
**Oral sex (n = 109)**							
**Never**	32	29,36%	19	1,00			
**Occasionally**	57	52,29%	41	1.75	0.70	4.36	0.114
**Regularly**	20	18,35%	18	**6.16**	**1.22**	**31.19**	**0.014**
**Anal sex (n = 100)**							
**Never**	67	67,00%	47	1,00			
**Occasionally**	26	26,00%	17	0,80	0,31	2,10	0,328
**Regularly**	7	7,00%	6	2,55	0,29	22,60	0,200
**Smoke (n = 117)**							
**Never**	33	28,21%	23	1,00			
**1-10 cigarettes a day**	25	21,37%	15	0,65	0,22	1,94	0,221
**11-20 cigarettes a day**	29	24,79%	21	1,14	0,38	3,44	0,407
**>20 cigarettes /day**	30	25,64%	28	**6,09**	**1,21**	**30,61**	**0,014**
**History of sexually transmitted diseases (STDs) (n = 111)**							
**no**	76	68,47%	41	1,00			
**yes**	35	31,53%	23	1,64	0,71	3,76	0,877
**Clamidia trachomatis**	2	5,71%	2,5	4,27	0,20	91,92	0,177
**herpes genitale**	6	17,14%	6,5	11,10	0,60	204,05	0,053
**Multiple sexually transmitted diseases**	27	77,14%	21,5	2,82	1,05	7,57	0,020
**History of genital warts (n = 117)**							
**no**	65	55,56%	52	1,00			
**yes**	52	44,44%	35	0,51	0,22	1,19	0,061

Statistically significant odds ratios are shown in bold. In cases where a frequency of 0 appeared, a correction was applied by adding 0.5.
